# Salicylic acid-mitigates abiotic stress tolerance *via* altering defense mechanisms in *Brassica napus* (L.)

**DOI:** 10.3389/fpls.2023.1187260

**Published:** 2023-07-25

**Authors:** Essa Ali, Sayed Hussain, Fazal Jalal, Muhammad Ali Khan, Muhammad Imtiaz, Fazal Said, Muhammad Ismail, Salman Khan, Hayssam M. Ali, Ashraf Atef Hatamleh, Munirah Abdullah Al-Dosary, Walid F. A. Mosa, Farooq Shah

**Affiliations:** ^1^ Institute of Plant Genetics and Developmental Biology, Zhejiang Normal University, Jinhua, China; ^2^ Department of Horticulture, Abdul Wali Khan University Mardan, Mardan, KP, Pakistan; ^3^ Department of Agronomy, Abdul Wali Khan University Mardan, Mardan, KP, Pakistan; ^4^ Department of Entomology, Abdul Wali Khan University Mardan, Mardan, KP, Pakistan; ^5^ Department of Botany and Microbiology, College of Science, King Saud University, Riyadh, Saudi Arabia; ^6^ Plant Production Department (Horticulture-Pomology) Faculty of Agriculture, Saba Basha, Alexandria University, Alexandria, Egypt

**Keywords:** abiotic stressors, antioxidant enzymes, mineral nutrients, oilseed rape, osmo-regulatory substances, photosynthetic pigments

## Abstract

Under the changing climate due to global warming, various abiotic stresses including drought (D) and salinity (S) are expected to further trigger their devastating effects on the already vulnerable crop production systems. This experiment was designed to unravel and quantify the potential role of exogenous application of salicylic acid (SA) in mitigating both D and S stresses and their combination (D+S), with three replications using CRD (Completely Randomized Design). The obtained results of the current study demonstrated significant effects of all three types of stresses (D, S, and D+S) on various parameters in *Brassica napus* plants. Quantifying these parameters provides a more informative and precise understanding of the findings. Current results revealed that all three stress types (D, S, and D+S) resulted in a reduction in leaf area (13.65 to 21.87%), chlorophyll levels (30 to 50%), gaseous exchange rate (30 to 54%) and the concentration of mineral ions compared to non-stressed plants. However, application of SA helped in mitigating these stresses by ameliorating the negative effects of these stresses. Moreover, Malondialdehyde (MDA) contents, an indicator of lipid per-oxidation and oxidative stress, the levels of antioxidants, proline content, an osmolyte associated with stress tolerance, and sugar content in the leaves were elevated in response to all stress conditions. In addition, the ultra-structures within the leaves were negatively affected by the stresses, while an application of SA considerably minimized the deterioration of these structures thus providing protection to the *brassica* plants against the stresses. In a nutshell, the findings of this study suggest that SA application in S, D and S+ D stresses provides evasion to the plants by improving different physiological and growth indices. The application of Salicylic Acid (SA) mitigated the negative effects of the stresses on all the above parameters, reducing MDA contents (47%), antioxidants (11 to 20%), proline (28%), sugar contents (20.50%), and minimizing the deterioration of ultra-structures. The findings emphasize the potential mitigatory role of SA in mitigating D and S stresses and highlight the need for further research to understand the underlying mechanisms in detail and explore its practical application in farming practices.

## 1 Introduction

Agriculture activities are closely associated with the existing atmosphere, with majority of crops often effects with different biotic and abiotic stresses. Common abiotic stress conditions which immensely hinder the normal growth and development of agriculture produce includes drought, salinity, waterlogging, cold, heat, and temperature fluctuations (Raza et al., 2019a and Raza et al., 2020a). Drought and Salinity are rated as the most drastic and deadly stress conditions, affecting about 26% & 20% of the agriculture acreage, respectively (USDA-FAO, 2018). Arid And semi-arid agricultural land is mostly subjected with the issue of salt stress (Sabagh et al., 2019). In the prevailing scenario of climate change, normal crop production is becoming challenging with every day passes on as various kind of abiotic stresses are get severe (Anderson et al., 2020; Zulfiqar and Hancock, 2020). The extent of damage caused due to drought and salt stress are very high and therefore they are considered as lethal among the list of all abiotic stress factors (Pokhrel et al., 2021). These abiotic stress factors has increased with every bit of human development as the anthropogenic activities in the era of progressive industrial development has caused serious damages to the agricultural system and its produce in form of introducing and enhancing various stress factors such as raised temperature, UV-B radiation, ozone depletion, metal/metalloids, water scarcity, salinity and nutrient depletion or excess (Anjum et al., 2014a). Abiotic stressors, including drought and salinity, alter their growth and development, seriously threatening crop productivity (Zhang et al., 2022). Both natural and anthropogenic stressors can lead to environmental challenges to plant growth, such as drought and salinity. Among all the environmental and climatic stress factors, these two abiotic-natured stresses (drought and salinity) are reflected as the most significant limiting factor towards crop productivity and growth enhancement, influencing about 40% and 11% of the world irrigated land, respectively (FAO et al., 2020). These abiotic factors can cause serious damages to the overall physiology and internal biology of a crop, for instance drought condition can cause wide range of biochemical and physiological changes in plang body and also carry damages its metabolic system (Abid et al., 2018; Qaseem et al., 2019). These environmental challenges pose a serious threat to agricultural production. Major grain producers and exporters such as China, India and the United States are currently experiencing severe water shortages in several important agricultural regions. A survey conducted by China’s Ministry of Water Resources found that during the 15.5-year plan, drought stress affected more than 25.67 million hectares of farmland annually, resulting in a reduction of up to 35 million tons of agricultural production and economic losses of up to about $35 billion (Ahmed et al., 2018).

In general, the simultaneous presence of multiple abiotic stresses has a more severe effect on agricultural production than a single stress situation (Mittler, 2006). Studies on crops such as potatoes (Levy et al., 2013), wheat (Yousfi et al., 2012) and barley (Yousfi et al., 2010) have shown that the simultaneous effect of saltiness and dryness on production is more in respect the effects of each individual stress. Previous studies have mainly convergent on the outcome of individual emphasis on plants (Wu et al., 2013), with tiny knowledge available on the physical and molecular mechanics that contribute to plant acclimatization to accumulation of saltiness and dryness stress (Mittler, 2006). However, modern research suggests that the consequence of plants to multiple physical stresses is distinct and may not be straight away generalized from the result of plants towards all stress separately (Rollins et al., 2013). Nature can evolve itself to the prevailing atmosphere changes, plants can overcome the drastic effect of drought and salt stress by activating a conducive defensive mechanisms in form of producing secondary metabolites, glycine betaine (GB), antioxidant enzymes, osmolyte-linked proline (PRO), carbohydrates, and total soluble proteins (TSP) (Shafiq et al., 2014; Shahzad et al., 2019). It was recorded that osmotic adjustment decreased the osmotic potential in plants and exhibited multiple benefits to plants in terms of enhanced growth patterns, adequate cell turgor pressure, and also modulates suitable metabolic activities inside a plants cell (Mafakheri et al., 2010). Furthermore, it was also recorded that glycine betaine immensely accumulates in maize plants while plants were subjected to water scare condition, which can increase all the growth traits (Gou et al., 2015). Similarly, proline acts as a conducive osmolyte during osmoregulation in water-stressed cotton plants (Habib et al., 2016). Plants develop different responsive-strategies towards various abiotic stresses, reflecting their variant levels of tolerance capacities (Noman et al., 2018; Ahmad et al., 2021). Plants subjected with water stress can adversely affect the biochemical, physiological and metabolic mechanisms in plants thus limiting their growth rate and development and quality of produce (Raza et al., 2020a). Along with drought and salt stress, the drastic influence of various other abiotic factors such as high and low temperature ranges (Du et al., 2016), waterlogging and metal toxicity (Lv et al., 2016) on crop quality and quantitative attributes have been reported in various crops, such as rapeseed (Raza et al., 2020a), and *Brassica napus* (Hatzig et al., 2014). To maintain yield on marginal land subject to stress, the most effective approach is to breed stress-tolerant crops. Therefore, it is crucial to determine inherited resources with higher permissiveness to nonbiotic stresses, particularly that occur at the same time in the piece of land, such as salinity and drought, and to realize their mechanics.

Plant hormones and growth regulators are integrally involved in modulating plant developmental systems and signal pathways, thus highlighting their pivotal role in responsive- strategies of plants towards various abiotic and biotic factors (Asgher et al., 2015). Enlisted to other integral plant hormones, Salicylic acid (SA) is basically a phenolic compound which can regulate the growth and developmental spectrums of plant and their response to different stress conditions (Khan et al., 2013b). SA is known for its involvement in regulating essential physiological and biological mechanisms in a plant body, worth mentioning such as photosynthesis, antioxidant defense responses, nitrogen metabolism, glycinebetaine (GB) and proline metabolism, thus strenthing defensive mechanism in plants against various abiotic stress factors (Miura and Tada, 2014).Beside its association in regulating defense-related genes and adequate resistance towards different biotic stress conditions (Kumar, 2014), SA has also been reported for its involvement in tolerance mechanisms of some major plants towards wide range of abiotic stress factors such as drought (Fayez and Bazaid, 2014), salinity (Khan et al., 2014; Nazar et al., 2015), metal (Zhang et al., 2015), and heat stress (Khan et al., 2013b) and osmotic (Alavi et al., 2014). Exogenous application of SA to plants in various ways, either by seed soaking method, nutrient solution, spray method or in general irrigation, can activate different mechanisms to better equipped plants with abiotic stress tolerance (Khan et al., 2013b; Palma et al., 2013; Khan et al., 2014).

Salicylic acid (SA) a plant’s hormone plays a vital role in regulating several physicochemical processes and have shown a significant impact on plants responses to overcome biotic and abiotic stress (Syeed et al., 2011). Although high concentrations of AS can generate oxidative stress and cell death in plants due to H_2_O_2_ accumulation, low concentrations of AS have been found to promote resistance to most of these stresses (Horvath et al., 2007).

Salt stress acclimatization in plants with SA improves through mechanisms such as ion exclusion and/or categorization, osmoregulation, reduction of membrane lipid peroxidation, protein kinase synthesis, and regulation of the antioxidant defense system (Khalifa et al., 2016; Yadu et al., 2017). However, the impact of exogenous application of SA on the plant stress tolerance habit is influenced by several factors, including the concentration, the mode of application and the general conditions of the plant, such as its stage of development, the oxidative balance of the cells and the previous state exposure to biotic or abiotic stress (Horvath et al., 2007). Salicylic acid (SA) is one of the pivotal phenolic compound produced adequately inside the plant body and regulates its various functions (Arif et al., 2020). Water-deficit conditions influence the endogenous biosynthesis of SA thus numerous studies has recommended the exogenous application of SA to plants which can improve its performance under unfavorable abiotic stress conditions (Abbaszadeh et al., 2020).

In present era, where the world is facing evolving with wide spectrum changes in urbanization, industrialization and climate change, crop production is threatened with various kinds of abiotic stresses and environmental hazards. Rapeseed (*Brassica napus* L.), just like other oil seed crops, are very sensitive to abnormal abiotic stress condition which will immensely reduce its growth patterns and also predominantly reduce its oil quality and quantity (Luo et al., 2017). Thus increasing tolerance capacity of rapeseed for various abiotic stress factors is the basic principal towards enhanced oilseed production (Luo et al., 2015; Raza et al., 2020a). The objective of this study is to explicate the physiological and biochemical processes of *Brassica napus* L. subjected to water- deficit an salt stress which will eventually lead towards its adaptation strategies to climate change. We will also investigate the influence of exogenous application of Salicylic Acid (SA), alone or in combination on the defense mechanism of *Brassica napus* L. towards drought and salt stress conditions.

## 2 Materials and methods

### 2.1 Plant’s materials and treatments

The current experimental study was carried out at the Faculty of Agriculture, The University of Poonch, Rawalakot, AJK, Pakistan, employing both hydroponic and field methods. The experimental area lies a Longitude 73° 45’ 34.93” E, Latitude 33° 51’ 32.18” N and an altitude of 1638 m. Oilseed rape seedlings were cultivated in pots using a mixture comprising 1/4 vermiculite, 1/2 compost, and 1/4 sand (v/v/v) for one month under controlled environmental conditions during the end of October. Subsequently, the seedlings were transferred to an experimental screen house to acclimatize to natural light conditions. Healthy seedlings with similar morphological characteristics were then transplanted into a container/pot of 5 L in volume having a Basal Nutrient Solution (BNS) of 4.5 L. After one week, different treatments were applied to the respective containers, including the control (BNS), drought stress (PEG) 10%, salinity stress (NaCl) 100 mM, control with Salicylic Acid (SA) 50 µM, combined drought and salinity stress (D + S), and SA with D + S stress. Salicylic acid (SA) was applied every 5th day until the harvest. Fifteen days after treatment, samples were collected from the treated plants to assess various parameters.

### 2.2 Leaf and seedling architecture analysis

Morphological parameters, including leaf area (cm2) and seedling height (cm), were measured using a leaf area meter (CID, CI-202) and a measuring scale, respectively (Gil-Ortiz et al., 2020).

### 2.3 Photosynthetic pigments and photosynthesis-related parameter analysis

A method previously described by Lichtenthaler (1987), was used to measure the contents of photosynthetic pigments (Chlorophyll-a and chlorophyll-b, mg/g FW) in fresh leaves of Brassica napus. After seven (7) days of treatment, fresh leaves were collected and 0.5 g of the collected leaves was grinded and mixed in a 10 mL tubes containing 80% acetone. The tubes were thoroughly mixed with shaker and then incubated for one night in the dark at room temperature. The extract concentration was determined using an ultraviolet spectrophotometer (UV-2100, UNIC, Shanghai, China) with the wavelengths of 665 nm and 649 nm respectively.

The parameters related to photosynthetic gas exchange, including leaf net photosynthetic rate (Pn, μmol m^-2^ s^-1^), internal CO_2_ concentration (Ci, μmol mol^-1^), stomatal conductance (gs, mol H_2_O m^-2^ s^-1^), and transpiration rate (Tr, mmol H_2_O m^-2^ s^-1^) were observed using an open type and portable photosynthesis system (LCA4. Bio-Scientific. Great Amwell. Herts. UK). The measurements were taken between 09:00 and 11:00 using the third top leaf. The CO_2_ concentration in the leaf chamber was maintained at 400 µmol mol^-1^, and the airflow speed was set at 500 µmol s^-1^. The photosynthetically active radiation (PAR) was maintained at 1000 µmol m^-2^ s^-1^. The air relative humidity was kept at 75 ± 5%, and the leaf temperature was maintained at 24 ± 2°C. The data were collected at intervals of 2-3 minutes with a minimum of three replicates.

### 2.4 Determination of osmo-regulatory substances analysis

The determination of malondialdehyde (MDA) content was conducted using a modified thiobarbituric acid (TBA) method (Kotula et al., 2020). Leaf samples (0.5 g) were homogenized with liquid nitrogen and 80% ethanol. The resulting mixture was centrifuged in 2 mL microcentrifuge tubes at 6000 rpm for 5 minutes. Aliquots of 0.7 mL of each supernatant were combined with 0.7 mL of 0.65% TBA in 20% trichloroacetic acid (TCA) and 0.01% butylated hydroxytoluene (BHT). Another set of 0.7 mL samples was mixed with 0.7 mL 20% TCA and 0.01% BHT. The microcentrifuge tubes were then incubated at 95°C for 25 minutes, followed by centrifugation at 6000 rpm for 5 minutes after cooling. The absorbance at 450 nm, 532 nm, and 600 nm was measured using a UV-Vis spectrophotometer (Evolution 210, Thermo Scientific), and the concentration of MDA (nM g^-1^ FW) was determined using an extinction coefficient of 157 mM cm^-1^. The content of proline was determined by a modified method based on Abdel and Tran (2016). Fresh shoots (0.5 g) were ground using a rapid automatic sample grinding instrument (JXFSTPRP24, Shanghai Jingxin Industrial Development, Shanghai, China) and digested in 5 mL of 3% aqueous sulfosalicylic acid. Afterwards, 2 mL of the extract solution was mixed with 2 mL ninhydrin reagent and 2 mL glacial acetic acid. The mixture was then boiled at 100°C for 60 minutes and cooled in an ice bath for 5 minutes. Subsequently, the mixture was extracted with 4 mL toluene, vigorously mixed using a vortex for 15-20 seconds, and cooled to room temperature. The free toluene was measured at 520 nm using a spectrophotometer (Beckman Coulter Inc., Fullerton, CA, USA). The amount of soluble sugar was determined by treating the sample with 80% ethanol according to the method described by Dubois et al. (1951). The sample was filtered, and the extract was heated in a water bath at 80°C for 1 h. Afterward, 2.5 mL of sulfuric acid (H_2_SO_4_) was added and mixed well. 0.5 mL of the mixture was mixed with 1 mL of 18% phenol, and the mixture was incubated at 37°C. The absorbance was recorded at 490 nm.

### 2.5 Quantification of antioxidant enzyme activities

To determine the enzyme activity, leaf samples weighing 0.5 g fresh weight (FW) were pulverized into a powdered form using a mortar and pestle. Subsequently, they were homogenized in 8 mL of 50 mM potassium phosphate buffer (PPB) with a pH of 7.8 under cooling conditions. The resulting homogenized solution was subjected to centrifugation at 10,000 rpm for 20 minutes at a temperature of 4°C, yielding a crude enzyme extract for the measurement of superoxide dismutase (SOD), peroxidase (POD), catalase (CAT), and ascorbate peroxidase (APX) activities (Hussain et al., 2015; Ahanger et al., 2018; Ahmad et al., 2020).

Superoxide dismutase (SOD; EC 1.15.1.1) activity was assessed by inhibiting the photochemical reduction of nitro blue tetrazolium (NBT). The reaction mixture consisted of 50 mM PPB (pH 7.8), 13 mM methionine, 75 mM NBT, 2 mM riboflavin, 0.1 mM EDTA, and 0.1 mL of the enzyme extract in a total volume of 3 mL. The unit of SOD activity was defined as the quantity of enzyme required to cause a 50% inhibition of NBT reduction, as measured at a wavelength of 560 nm.

For peroxidase (POD; EC 1.11.1.7) activity determination, 0.1 mL of the enzyme extract was combined with 50 mM PPB (pH 7.0), 1% (m/v) guaiacol, and 0.4% (v/v) H2O2. The absorbance was quantified at a wavelength of 470 nm.

Catalase (CAT; EC 1.11.1.6) activity was measured by employing H_2_O_2_ (with an extinction coefficient of 39.4 mM-1 cm-1). A reaction mixture of 3 mL was prepared, comprising 50 mM PPB (pH 7.0), 2 mM EDTA-Na2, 10 mM H_2_O_2_, and 0.1 mL of the enzyme extract. The measurement was performed at a wavelength of 240 nm.

Ascorbate peroxidase (APX; EC 1.11.1.11) activity was determined using a reaction mixture (3 mL) containing 100 mM phosphate buffer (pH 7), 0.1 mM EDTA-Na2, 0.3 mM ascorbic acid, 0.06 mM H_2_O_2_, and 0.1 mL of the enzyme extract. The change in absorption was monitored at 290 nm for a duration of 30 seconds after the addition of H_2_O_2_.

### 2.6 Ultrastructural analysis using transmission electron microscopy

Small leaf fragments, measuring approximately 1 mm^2, were collected from the fully expanded topmost leaves of the plant at 10 days after treatment (DAT) for the purpose of investigating intracellular ultrastructures. The samples were subjected to analysis using a JEOL TEM-1230EX transmission electron microscope. Initially, the leaf specimens were fixed overnight in a 2.5% glutaraldehyde solution (v/v) and subsequently washed three times with a 0.1 M Sodium Phosphate Buffer (SBP) at pH 7.0. Post-fixation was carried out by treating the specimens with 1% osmium tetraoxide (OsO_4_) for 1 hour, followed by washing with a 0.2 M Sodium Phosphate Buffer (SPB) at pH 7.2 for 1-2 hours. Dehydration of the samples was performed using a series of graded ethanol concentrations (50, 60, 70, 80, 90, 95, and 100%) and 100% acetone. The samples were then infiltrated and embedded in Spurr’s resin. Thin sections with a thickness of 80 nm were prepared, mounted on copper grids, and observed under a JEOL TEM-1230EX transmission electron microscope operating at an accelerating voltage of 60.0 kV.

### 2.7 Analysis of mineral nutrient content

Approximately 0.1 g of oven-dried shoot and root tissues, subjected to drying at 65-70°C for 72 hours, were pulverized to determine the concentrations of essential mineral nutrients, namely Magnesium (Mg), Calcium (Ca), Zinc (Zn), and Iron (Fe). Mineralization was performed by heating 5 ml of 65% nitric acid (HNO_3_) using a microwave digester (Microwave 3000; AntoonPaar). The volumes of the digested samples were adjusted to 10 ml by adding Milli-Q water. Subsequently, the specimens were analyzed using an inductively coupled plasma-optical emission spectrophotometer (ICP-OES; Optima 8000DV; PerkinElmer) to quantify the content of mineral nutrients.

### 2.8 Statistical analysis

Analysis of variance (ANOVA) was presented on the obtained data for various parameters, and appropriate completely randomized design was used for testing the treatments. Tukey test was used to calculated the probability level greater than 0.05. The software OriginPro7 was utilized to generate the figures.

## 3 Results and discussions

### 3.1 Leaf and seedling architecture analysis

**Figure 1:**
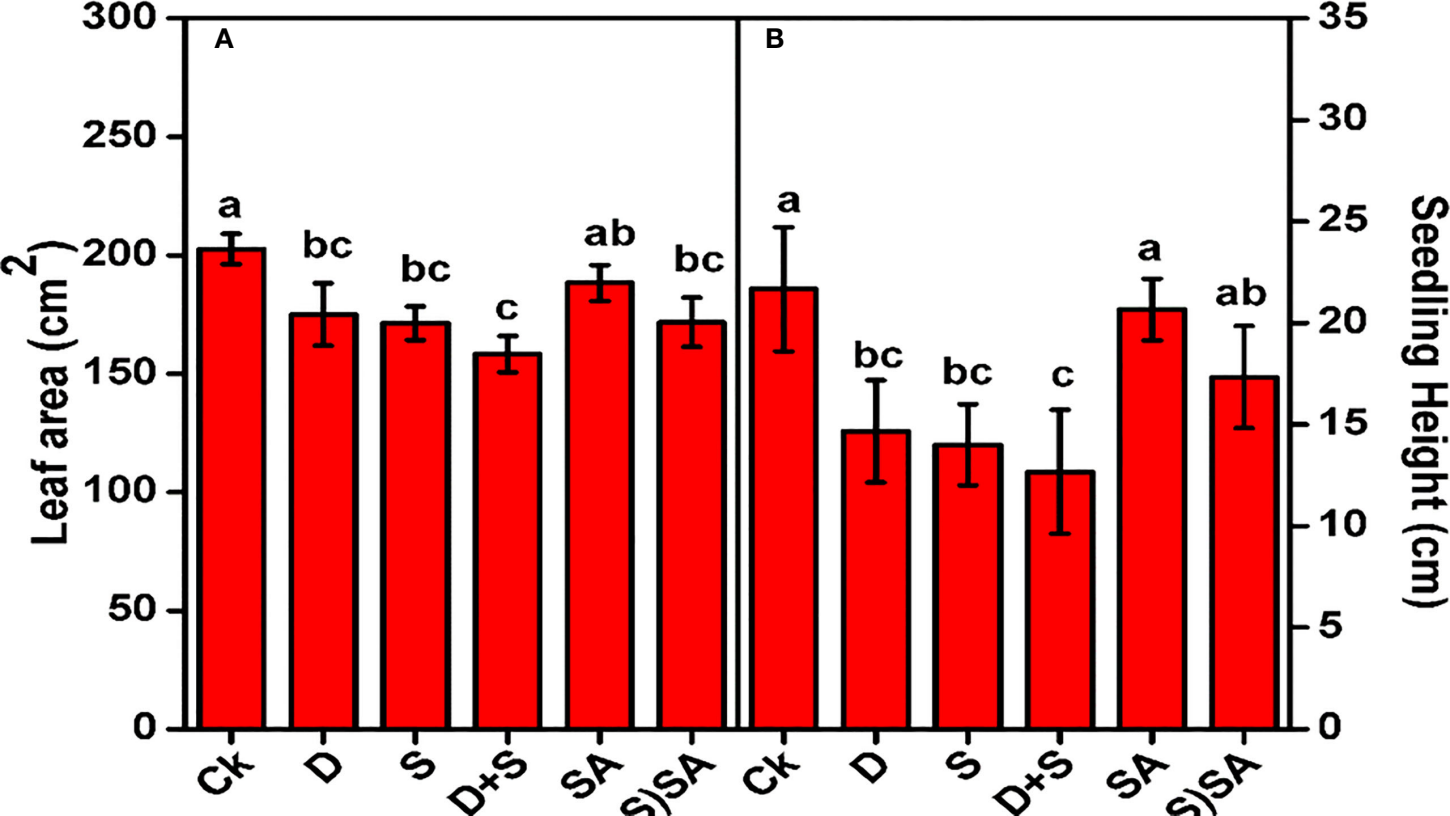
Effect of Drought, Salinity, and SA on leaf area **(A)** and seedling height **(B)** of *Brassica napus* genotype. Statistical differences at a 5% level exist between means labeled with different letters (a-e) for each attribute (mean ± SD; n = 3).

**Figure 1** illustrates the influence of drought and salinity stress alone and in combination on the shoot length and leaf area of *Brassica napus* plants. Both stress conditions exhibits a considerable decrease in shoot length and leaf area, with more prominent effect reflected in combine stress growing condition. Drought stress decreases the shoot length by (32.2%) and leaf area by (13.6%). Similarly, Shoot length and leaf area declined in salt stress by (33.4%) and (15.4%). Combined effect of both the stresses (D+S) results in severe decrease in shoot length (41.5%) and leaf area (21.8%) as compared to non-stressed plants. However, drought and salinity stressed-subjected plants while treated with SA showed enhanced shoot length (26.9%) and leaf area (7.7%) as compared to non-SA treated plants (**Figure 1**). This response showed that SA application could effectively improve *Brassica napus* plants growth under water and salinity stress. In current study, the decline growth pattern of *Bassica napus* L. seedlings under water-deficit and salinity was probably due negative affect on important metabolic functions linked with photosynthesis, chlorophyll biosynthesis rate, oxidative metabolism and cell division & enlargement. Cellular elongation and differentiation process drastically slow-down under salt and drought stress conditions (Abid et al., 2018; Sarabi et al., 2019). Also, vegetative growth features and traits of major crops considerably reduce under water-deficit stress (Damalas, 2019). Moreover, the retardant impact of water and salt stress on brassica napus plants were mitigated with SA application, with partial recovery in shoot and leaf growth and also boosting the photosynthesis synthesis rate and chlorophyll content leaves (Saudy et al., 2023b; Shaaban et al., 2023). Similar findings were also reported in various other crop species, including sweet basil (Damalas, 2019), maize (Bijanzadeh et al., 2019), sesame (Najafabadi and Ehsanzadeh, 2017), sunflower (Kosar et al., 2018), and squash (El-Mageed et al., 2016). Our results supplements the idea that SA is playing apivotal role in defense mechanism and protecting the photosynthetic apparatus in brassica plants (Kosar et al., 2018), with mitigating the photosynthetic and chlorophyll activities and thus encouraging overall plant growth traits in water deficit and saline stress conditions.

### 3.2 Photosynthetic pigments and photosynthesis-related parameter analysis

**Figure 2:**
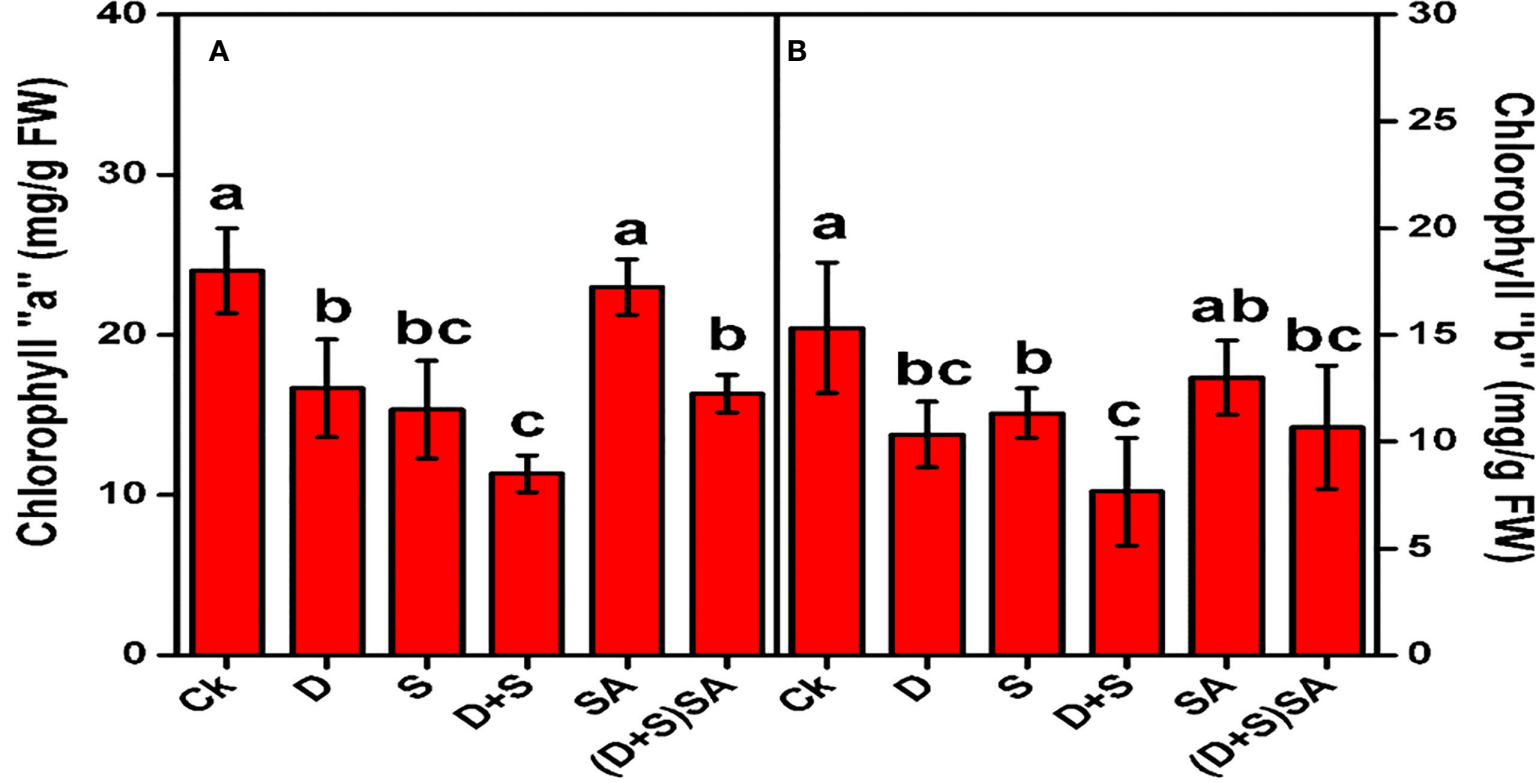
Effect of Drought, Salinity, and SA on Chlorophyll “a” **(A)** and Chlorophyll “b” **(B)** of *Brassica napus* leaves. Statistical differences at a 5% level exist between means labeled with different letters (a-e) for each attribute (mean ± SD; n = 3).

Drought and salt stress both individually and in combination predominantly affect the photosynthetic pigments in *Brassica napus* plants (**Figure 2**). Drought stress drastically reduced Chl-a contents by (30.5%) and Chl-b contents by (32.6%) as compared to plants without stress. Similarly, salt stress condition also decreases Chl-a by (36.1%) and Chl-b by (26%). Moreover, the combined impact of D+S depicts drastic decline in concentrations of Chl-a (52%) and Chl-b (49%). However, brassica plants subjected with SA predominantly elevated the photosynthetic pigments Chl-a (29%) and Chl-b (28%), as compared with non-SA treated plants (**Figure 2**). A reduction in chlorophyll content reflects as a characteristic indication of oxidative stress and may be due to the pigment photo-oxidation and chlorophyll degradation under drought stress environments. Decline in concentration of Chlorophyll pigments under water deficit condition might be due to disruption in chlorophyll biosynthesis which can decrease the chlorophyll contents in leaves (Ashraf and Harris, 2013), and thus cause a cosiderbale decline in photosynthesis rate in plants (Amirjani and Mahdiyeh, 2013). Similar kind of findings where a substantial decline in chlorophyll content and photosynthesis rate were also observed in wheat (Habib et al., 2020) and cucumber (Naz et al., 2016) grown under drought stress condition. Moreover, Ahmed et al. (2013b) and Saudy et al. (2021) also found a noticeable reduction in the chlorophyll contents (Chl-a and Chl-b) in barley plants raised in drought and salinity stress environment. The beneficial influence of SA in relation to the damaging effects of water-deficit and salt stress on production of chlorophyll pigments in brassica plants as displayed in **Figure 2**, could be accredited to its regulatory effects on Rubisco activity, and thus eventually, to enhance the photosynthesis process. SA probably plays its role in the Cellular membrane defense mechanism and also in scavenging the toxic ROS species produced during the oxidative stress condition. Another supplementary effect of SA could be attributed to stimulating the biosynthesis of protein kinases which plays a pivotal role in regulating cell division process at different stages of cell development (Ali et al., 2013). Few other reports further describes that photosynthesis process and stomatal conductance elevates due to SA treatment under growing condition prone with various abiotic stresses (Kosar et al., 2018). Similar kind of impact created by SA treatment was also recorded in *Triticum aestivum* L. (Maghsoudi et al., 2019).

### 3.3 Gas exchange parameters

**Figure 3:**
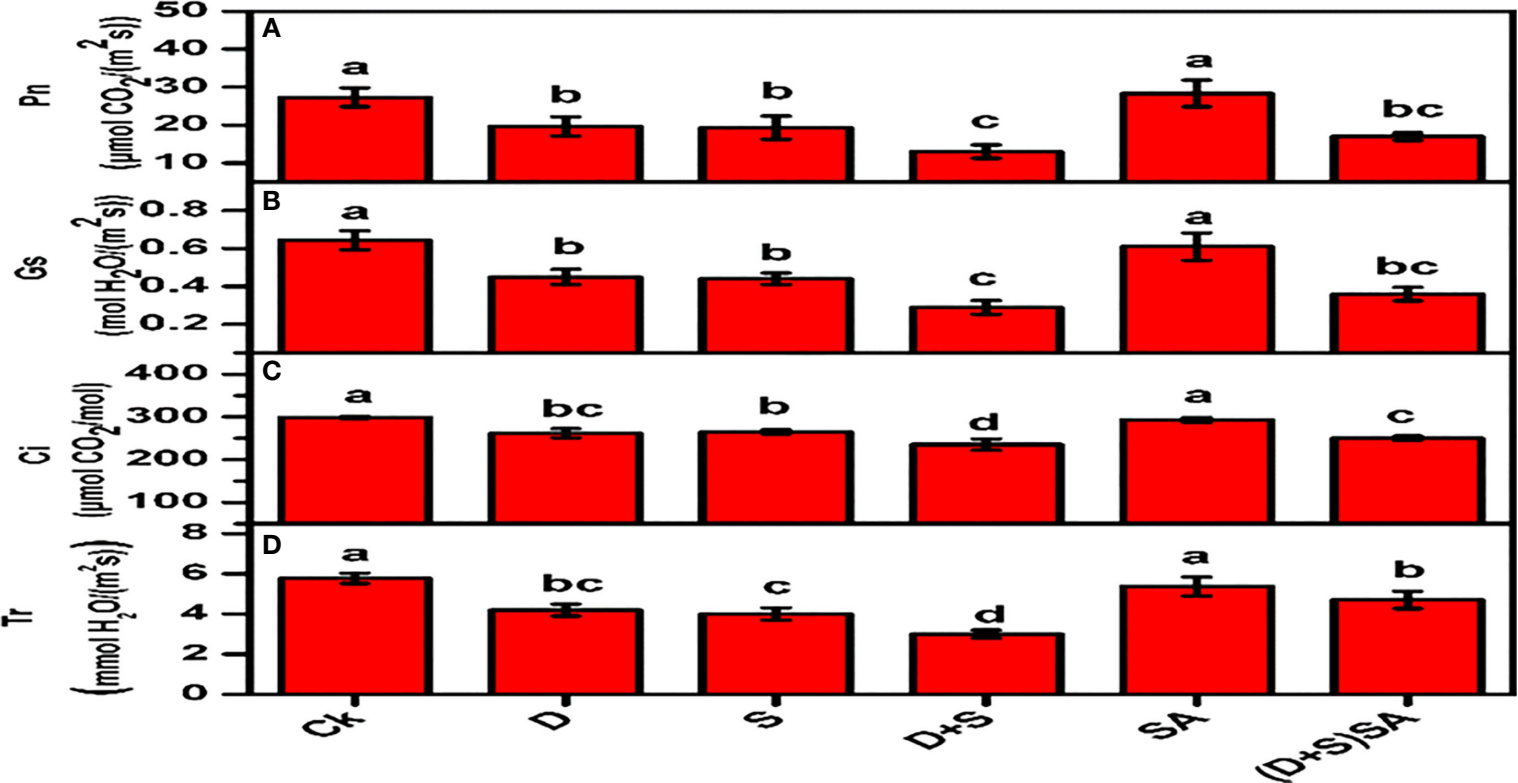
Effect of Drought, Salinity and SA on gas exchange parameters; Photosynthetic rate (Pn) **(A)**, stomatal conductance (Gs) **(B)**, internal CO_2_ concentration (Ci) **(C)**, and transpiration rate (Tr) **(D)** in leaves of *Brassica napus* genotypes. Statistical differences at a 5% level exist between means labeled with different letters (a-e) for each attribute (mean ± SD; n = 3).

The results presented in **Figure 3** depict the impact of water deficit and saline stress alone or in combination on all the traits associated with photosynthesis. Both the stress conditions significantly reduce various kinds of leaf exchange characteristics in *Brassica napus* plants including Pn, Ci, Tr, and Gs. Drought stress cause a decreases by Pn (28%), Ci (12.4%), Tr (26.3%) and Gs (29.6%) as compare to non-stress plants. Likewise, a declining pattern for Pn (29.2%), Ci (11.3%), Tr (29.8%) and Gs (31.6%) due to salt stress. Combined effect of D+S was severe and reflected with much declined values of leaf exchange catalyses’ Pn (52.4%), Ci (21.2%), Tr (47.3%) and Gs (54.3%) as compare to non-stress plants. However SA applied to plants subjected to combined stress condition can significantly increase all the aforementioned photosynthetic parameters such as Pn by (23.5%), Ci (6%), Tr (36.1%) and Gs (19.4%) as compare to its influence of control plants. These results indicate that SA can restore the negative effects of stress on plant overall growth and development. Both drought and salinity stresses have a significant negative impact on photosynthesis, which is among the initial step towards biological degradation of plant physiology and vitality (Sarabi et al., 2019). These effects may be direct, for instance lower CO_2_ availability due to limitations in diffusion through stomata and mesophyll or possible changes in photosynthetic metabolism, or they may depict as secondary effects such as oxidative stress. Drought among all the abiotic stress factors drastically stimulates the photo-oxidative degradation of photosystems in various plants (Hu et al., 2018), thus causing a decline in Pn concentration (Shao et al., 2016). Our findings also reflected that water deficit stress results also degraded the cellular membrane system in *Brassica napus* plants and caused decline in various leaf exchange characteristics Pn, Ci, Tr, and Gs. Disruption and ineffectiveness in chloroplast membrane system, due to swelling and damages caused to the thylakoid membranes (either stromal or granal) were observed in wheat and maize under water deficit stress factors. Salt stress is subjected with a significant decline in photosynthetic rate in many plant species, which may be due to decreased stomatal conductance, decline in photochemical capacity, and reduce metabolic processes during carbon uptake (Pérez-López et al., 2012). In another study, a predominant decline in transpiration rate, photosynthetic rate, stomatal conductance and inter-cellular CO_2_ were recorded for maize plants grown in water stress condition (Anjum et al., 2011). Moreover, numerous reports have reported that exogenously application of SA retained the membrane integrity of chloroplast & thylakoid structures in plants confronted with drought stress (Hu et al., 2018). In support with these earlier reports, our findings also depicted that exogenous application of SA stabilizes the integrity of cellular membrane structures, reducing the Pn, Ci, Gs, and Tr concentrations, and decreases the electrical conductivity in leaves of Brassica napus plants during both water-deficit and saline stress conditions (**Figure 2**).

### 3.4 Lipid peroxidation (MDA)

**Figure 4:**
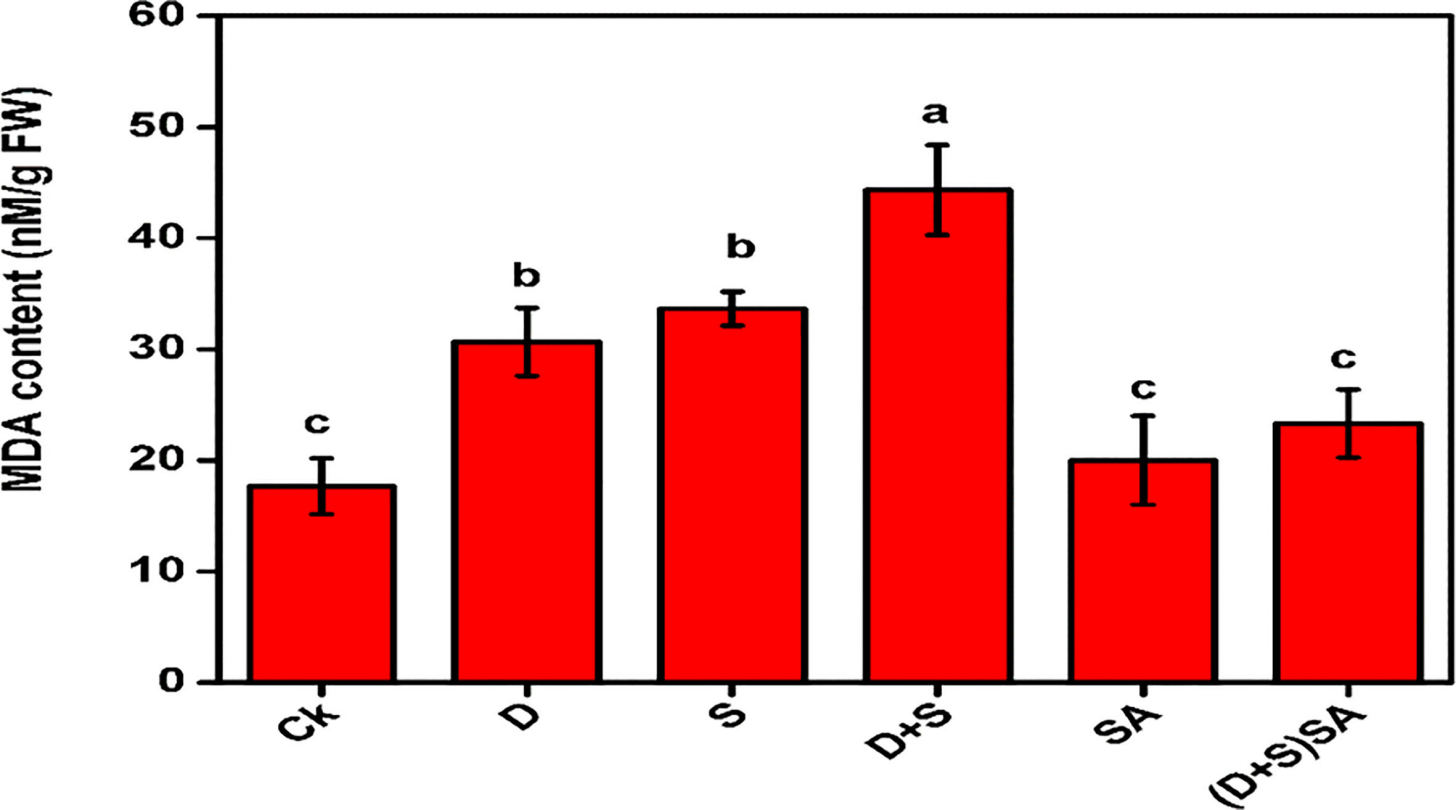
Effect of Drought, Salinity and SA on MDA content in leaves of *Brassica napus* genotypes. Statistical differences at a 5% level exist between means labeled with different letters (a-e) for each attribute (mean ± SD; n = 3).

An increase in ROS accumulation is a major consequence of drought and/or salinity stress conditions in plant cells. The application of salinity, dearth, and D + S treatments triggered a major rise in MDA production in the leaves of Brassica napus as compared to untreated plants (**Figure 4**). Water deficit and salinity stress caused an elevation in MDA by 42.3% and 47.5%, respectively. The impact was more pronounced in the D + S treatment increasing by 60.1% as compare to either drought or salinity stress alone and control plants. The application of SA caused a decrease in MDA content by 47.3% in B. napus leaves under D + S stresses as compare to plants without SA treatments (**Figure 4**). Water deficit-stress condition results in ROS accumulation due to decreased light absorption and minimum photosynthetic electron transport, which can cause photo-oxidative damages to the photosystems (Dalal and Tripathy, 2018; Yudina et al., 2020). Oxidative stress markers including H_2_O_2_, Electrolyte leakage, and MDA are found elevated in plants during abnormal environmental condition (Shao et al., 2016). Brassica napus plants leaves subjected to salt and water stress displayed an elevated levels for MDA concentrations as compare to non-stress plants. Our findings were similar to reports studied in *E. globulus* (Jesus et al., 2015) and *Carthamus tinctorius* L. (Bijanzadeh et al., 2019) and. Also, application of SA reduced the MDA concentrations in *Brassica anpus* L. plants grown in saline and drought- stressed conditions, reflecting that SA mitigated the oxidative stress inked with these abiotic stress conditions. Similar kind of findings were also reported for *Brassica rapa* L. (La et al., 2018) and *Triticum aestivum* L. (Maghsoudi et al., 2019), where MDA concentration and electrolyte leakages and H_2_O_2_ displayed a declining patterns with introduction of SA under saline and drought stress. Another study also recorded a novel feature in SA-subjected sweet basil plant that the relative water content (RWC) was also elevated in leaves, indicating that SA increases the membrane integrity during stress conditions (Damalas, 2019).

### 3.5 Antioxidant enzymes activities

**Figure 5:**
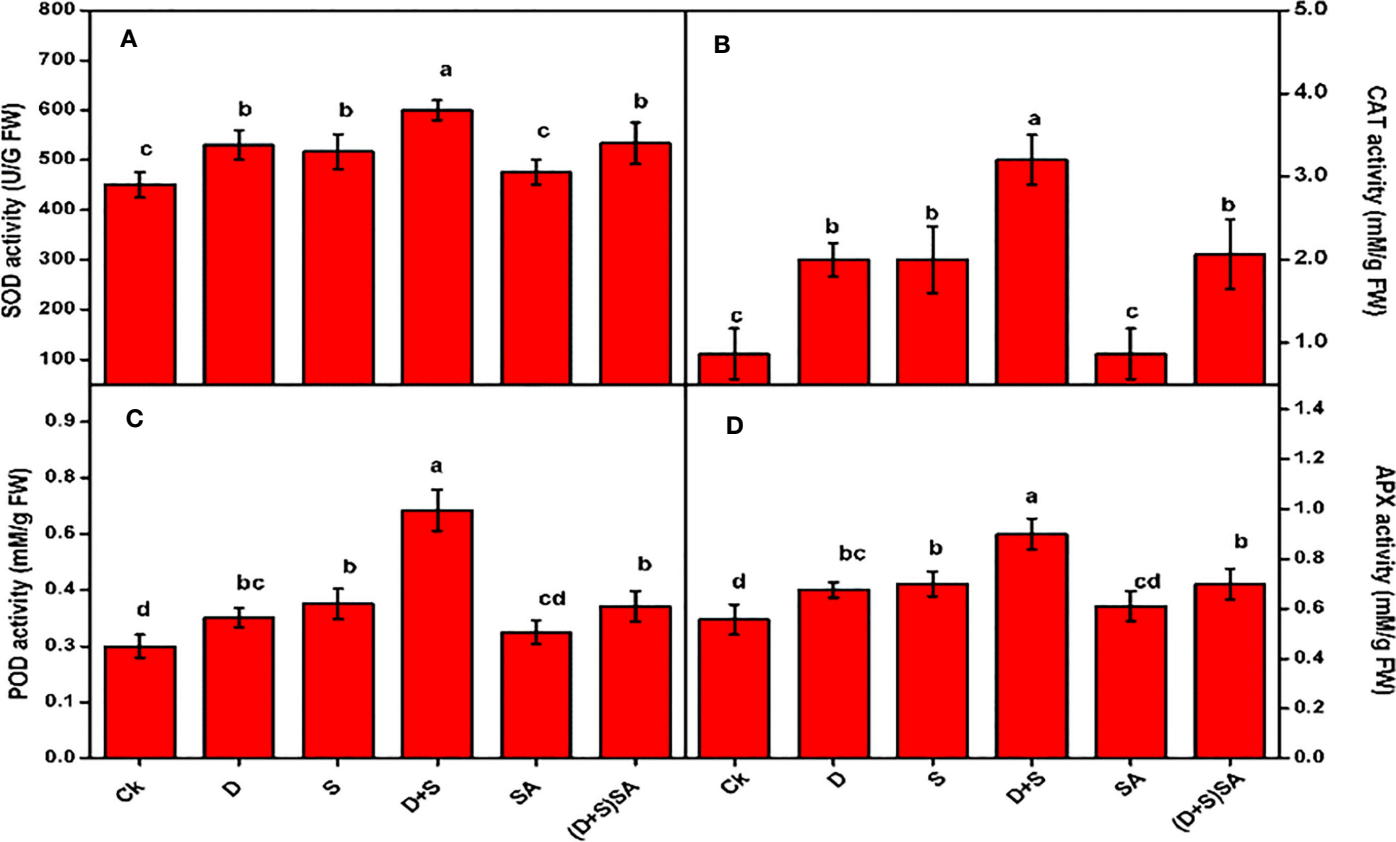
Effect of Drought, Salinity and SA on the activities of Antioxidant Enzymes in leaves of *Brassica napus* genotype; activities for SOD **(A)**, CAT **(B)**, POD **(C)** and APX **(D)** were measured separately. Statistical differences at a 5% level exist between means labeled with different letters (a-e) for each attribute (mean ± SD; n = 3).

A substantial raise in the concentrations of various antioxidant enzymes SOD, POD, APX and CAT were recorded for *brassica napus* plants while confronted with water-deficit and salt stress (**Figure 5**). The Combined influence of both the stress factors (D+S) was further severe as compare to single stress condition. Drought stress induced CAT, SOD, POD and APX activities by 57%, 15%, and 20.3%, 17% respectively, compared to well-watered brassica plants. Similarly, salinity stress also exhibited elevation in CAT (57%), SOD (12.7%), POD (26.8%) and APX (20.5%) as compared to unstressed (**Figure 5**). Similarly, combined stress (D+S) influence was highly significant with CAT (73%), SOD (25%), POD (54.5%) and APX (38.2%) increasing concentrations as contrast to control. However, when leaves were treated with SA, all the enzyme activities were found to decrease. Foliar application of SA decreases CAT, SOD, and POD activities by 35.4%, 11.1%, 17.4% and 22.2% respectively, as compared to non-SA treated plants (**Figure 5**). Reports confirm that water-deficit conditions leads to abnormal and harmful effects in plant body, majorly distortion of cellular membrane and structures, ion-leakages, inactivation of enzymes, and decreased osmotic regulation, due to increasing ROS accumulation inside infected plant cells (Shafiq et al., 2014). In present study, the rising activities of antioxidant enzymes in brassica napus plants due to drought and salt stress conditions can be linked to the activation of plant defense mechanism in order to safeguard plants against water deficit-induced ROS accumulation. The activation of antioxidant systems is a robust process in order to counter the oxidative stress under stress conditions, which involves different kind of enzymes mainly superoxide dismutase (SOD), peroxidase (POD), catalase (CAT) and ascorbate peroxidase (APX), ascorbate peroxidases and few non enzymatic molecules i.e phenols, glutathione, osmolytes and ascorbates (Cirulis et al., 2013; Ali et al., 2018). In this study, we observed the instance activation of these antioxidant enzymes in response to salt and water stress and further decrease with SA application. Similar results were reported earlier in different crops, with activation in antioxidants enzymes such as SOD, POD and CAT were observed under drought stress in grapes (Wang et al., 2014). Furthermore, several more studies further elaborated that SOD plays an integral role during detoxification, whereas enzymes such as CAT and POD were engaged in countering numerous latent oxidants in order to restore stress tolerance (Ashraf, 2009; Akram et al., 2018). An important indicator for plant stress tolerance is the up-regulation and activation of antioxidant enzymes during stress conditions (Yadav et al., 2016). The accumulation of antioxidants helps in minimizing the adverse effects of oxidative stress and to normalize the metabolic activities under stress condition. SOD converts O_2_^−^ to H_2_O_2_, which is further converted to water by ascorbate peroxidase (APX) utilizing ascorbate as an electron donor (Scandalios, 2005). Higher plants, when confronted with drought stress exhibits an increased buildup of antioxidant enzymes, including SOD, POD, CAT and APX, which protect against oxidative injury (Ozkur et al., 2009). In contrast, Yang et al. (2010) reported that at 25% field capacity, the activities of CAT, POD, SOD, GR and APX were elevated relative to 100% field capacity. In another study, Ozkur et al. (2009) reported opposite trends in activities of SOD, POD, CAT, GR and APX in response to NaCl stress condition in H. marinum and *H. vulgare* plants Several reports reflected that exogenous SA induction alleviate the activation of different enzymatic antioxidants especially during salt and water-deficit stress-induced *Brassica rapa* (La et al., 2019), safflower (Bijanzadeh et al., 2019) and wheat (Maghsoudi et al., 2019).

### 3.6 Buildup of proline and soluble sugars

**Figure 6:**
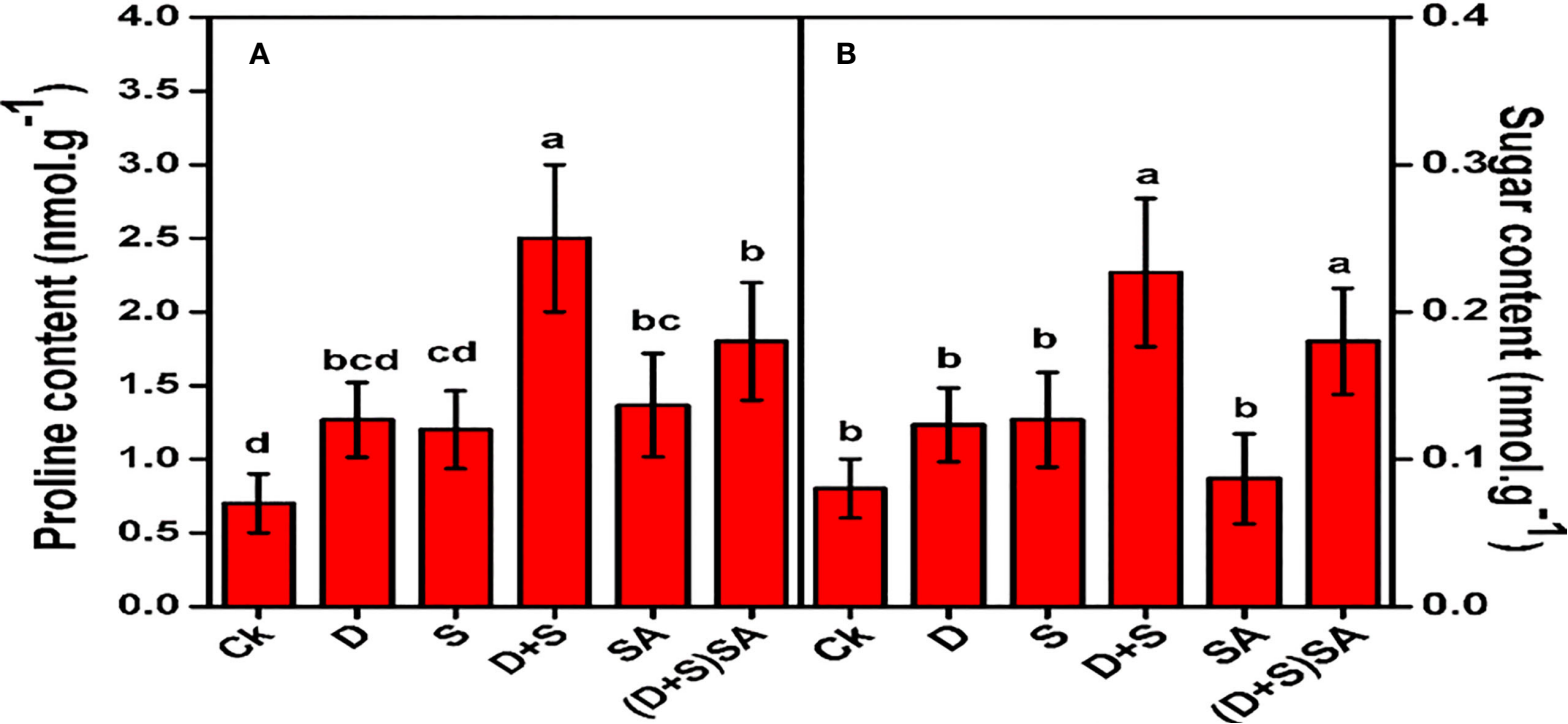
Effect of Drought, Salinity and SA on Proline **(A)** and sugar content **(B)** in leaves of *Brassica napus* genotype. Statistical differences at a 5% level exist between means labeled with different letters (a-e) for each attribute (mean ± SD; n = 3).

**Figure 6** illustrates that proline and soluble sugar levels in *Brassica napus* leaves were considerably increased under D + S stress conditions as compared to control. Drought stress elevated the leaf Proline and soluble sugars concentrations by 44.7% and 34.9% respectively, as compare to the plants without stress. Likewise the proline and soluble sugar in salt stress situation also raised by 41.6% and 28.7%, respectively. Similarly, Combine stressed effect (D+S) reflected a much higher elevation of 72% in proline and 64.6% in soluble sugars as compare to non-stressed plants. However, when SA was applied, the levels of proline decreased under D + S stress conditions, though no substantial effects were found in sugar contents (**Figure 6**). Foliar application of SA decreases Proline concentration by 28% and soluble sugar concentration by 20.5% relative to unsprayed stressed plants. Leaf free Proline and soluble sugars are the multifunction molecules found in plants that protect the plant cellular membranes against oxidation and dehydration conditions (Per et al., 2017; Annunziata et al., 2019). In present experiments, we will found an elevated pattern of proline for *Brassica napus* plants subjected to drought and saline stress conditions. Proline has been known for its multiple regulatory functions especially during abnormal growing conditions, with more profound roles are to safeguard the photosynthetic system, cellular membranes and enzymatic regulations, prevent cellular dehydration and act as a molecule. Therefore, proline may be closely linked with stress-tolerance mechanism in plants mainly protecting the cellular structures and regulating physiological processes inside plant body. Furthermore, Pro also decreases the oxidative stress in various plants by alleviating the activities of ROS-scavenging antioxidants which will reduce the accumulation of ROS species (Ibrahim and Abdellatif, 2016). Compatible solutes are typically small in size and easily dissolve in water, and they are generally considered safe even when found in high concentrations within a cell. Plants collect compatible solutes, including Proline and sugars, in order to aid in water absorption as a response to drought and salinity conditions (Ashraf and Foolad, 2007; El-Metwally and Saudy, 2021; El-Metwally et al., 2022). Apart from regulating osmotic pressure, these compatible solutes have been advised to play a key character in shielding cells from ROS accumulation that occurs during stress conditions. While K+ is the primary contributor to osmotic adjustment during the primary phases of stress, molecules like Pro, GB and glucose become more important during later stages (Nio et al., 2011; El-Metwally et al., 2021). In the present study, Pro and soluble sugars displayed elevation in *brassica napus* plants subjected to water and salt stress, and these levels decreased further after SA treatments alone or in combination. In another study, sweet basil (Damalas, 2019) and maize (Bijanzadeh et al., 2019) were reported for enhanced Pro and GB levels after SA treatments under water-deficit stress.

### 3.7 Accumulation of nutrient elements

**Table 1** illustrates the influence of individual and combination of both drought and salinity stress condition on the concentration of nutrient elements. Both drought (D) and salinity (S), either single or in combination, significantly reduced the levels of Ca, Mg, Fe, and Zn. Drought stress decreases the mineral concentration by Ca (40.6%), Mg (27.6%), Fe (34.4%) and Zn (28.1%) as compare to unstressed plants. Similarly, mineral conc. for Ca (36.3%), Mg (33.5%), Fe (26.5%) and Zn (23.1%) also reduced in saline stress condition. Combined influence of D+S was immense and cause a decrease Ca conc. by (53.7%), Mg (54.8%), Fe (14%) and Zn (43.7%) as contrast to unstressed plants. However, when SA was administered alone or in combination with salinity and drought, it resulted in an increase in the levels of the aforementioned elements, Ca (19.4%), Mg (20.6%), Fe (40%) and Zn (36%) as compare to non-SA applied plants, indicating the beneficial effects of SA during stressful conditions (**Table 1**). Limited water availability due to drought and salinity generally results in a reduced uptake of nutrients and lower tissue concentrations in crops (Saudy and El-Metwally, 2019; Mubarak et al., 2021; Salem et al., 2021; Abd–Elrahman et al., 2022). One of the most notable effects of water scarcity is on the capacity of roots to soak up nutrients and their mobility to upper portion of the crop/plant as shoots (Farooq et al., 2009; Saudy et al., 2020; Salem et al., 2022). In another study it was reported that drought and salinity stress predominantly reduce the Zn, Fe, and Mn content found in shoot and roots of Linseed plants and further that SA application could regulate this declining patterns (Mohsen et al., 2019). SA plays very important and necessary function in regulating various nutrient content inside the plants under different various stresses conditions (Khan et al., 2010). For instance, the application of SA enhanced the inhibition of Calcium (Ca), Zinc (Zn) and Magnesium (Mg) absorption caused due to excessive Manganese (Mn) in and important vegetable as cucumber (Shi and Zhu, 2008), while it also mitigated the Cd-induced inhibition of Calcium (Ca), Magnesium (Mg) and Iron (Fe) absorption in Linum usitatissimum (Khan et al., 2010).

**Table 1:** Effect of drought (D), salinity (S), and SA on mineral nutrients; calcium (Ca), magnesium (Mg), zinc (Zn), and iron (Fe) in the leaves of *Brassica napus* genotypes.

	Ca (mg/g DW)	Mg (mg/g DW)	Fe (mg/g DW)	Zn (mg/g DW)
**Control**	60.7 ± 4.0 a	12.5 ± 0.5 a	0.29 ± 0.04 a	0.32 ± 0.03 a
**Drought(D)**	35.3 ± 4.5 cd	9.1 ± 0.4 b	0.20 ± 0.02 bc	0.23 ± 0.04 bc
**Salinity(S)**	38.7 ± 7.8 c	8.3 ± 1.5 bc	0.21 ± 0.04 bc	0.25 ± 0.02 b
**D+S**	28.0 ± 3.0 d	5.7 ± 0.6 d	0.15 ± 0.03 c	0.18 ± 0.04 c
**SA**	64.3 ± 3.1 a	13.7 ± 1.5 a	0.31 ± 0.04 a	0.35 ± 0.03 a
**(D+S)SA**	49.0 ± 4.6 b	7.1 ± 0.4 cd	0.25 ± 0.04 ab	0.29 ± 0.04 ab

mean ą SD; n = 3.

### 3.8 Ultra-morphology of plants

**Figure 7:**
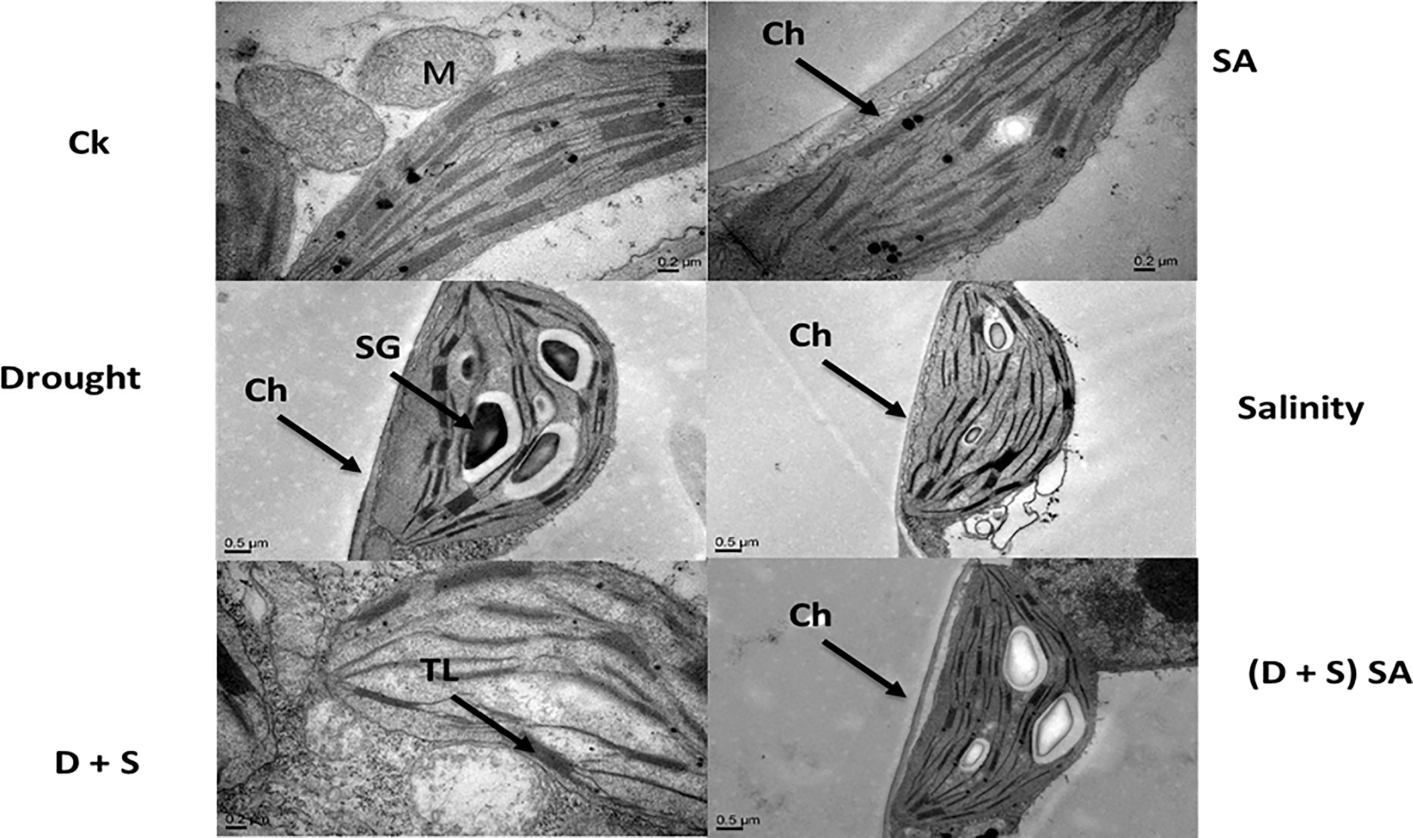
Effect of Drought, Salinity, D +S, and SA on ultra-structures of *Brassica napus* leaves.

In current study, the salt and water deficit stress resulted in obvious changes to the shape and size of chloroplasts in *Brassica napus* L. plants (**Figure 7**). Image further showed that the Chloroplast for *Brassica napus* plants subjected with stress conditions became oblate and spherical. Also an irregular and deformed shape of stroma thylakoids was observed, with grana lamella, and teas and faults decrease due to salt and drought stress (**Figure 7**). Chloroplast is the main power house for Photosynthesis process and the photoreaction is present inside the internal chloroplast membranes i.e., thylakoid. Drought and salt stress can disturb the sensitive formation of chloroplasts, destabilization of pigment-protein complexes, and breakdown of chlorophyll molecules, while also affect both quantity and makeup of carotenoids (Amirjani and Mahdiyeh, 2013). Our results are parallel to another study conducted by Ma et al. (2017) indicating that increasing abiotic stress levels especially salt stress in *Dianthus superbus* L. can induce the deformation in chloroplast size and shape, resulting into a oblate and spherical shape chloroplast, irregular grana with shrinked lamella and distorted thylakoid membrane structure. Another study reported that osmiophilic globules were found plastoglobuli in chloroplasts and linked with membrane breakdown, aging or senescence and may be stress factors (Lichtenthaler, 2013). Our results are also supported by another study reporting that saline stress may results in elevated ROS (H_2_O_2_, O_2_^−^) accumulation and damage the thylakoids membrane, thus reducing the photosynthetic rate (Ma et al., 2017). Elevated levels of NaCl lead to swelling of thylakoid membranes and reduction in chlorophyll fluorescence in the barley seedlings (Zahra et al., 2014). Both organelles, chloroplasts and mitochondria, are influenced differently in different cultivars under all given treatments. The drought-resistant wheat cultivar Katya demonstrates improved organelle preservation as compared to the sensitive variety Sadovo. Some studies have suggested that salicylic acid can alleviate stresses in different crops (Ali et al., 2018).

## 4 Conclusion

Based on findings of the study, it is concluded that applying Salicylic Acid (SA) during Salinity (S) and drought (D) stress conditions alone and their combinations (Salinity Drought) can help *B. napus* plants avoid damage by enriching different physiological as well as growth elements. The results on current study also convey important implication as they exhibit vigorous perspectives of SA applications while alleviating the ill-effects of D and S stress conditions. Further research is fortified to explore the specific utilizations involved and discover how SA can be applied in different farming practices.

## Data availability statement

The raw data supporting the conclusions of this article will be made available by the authors, without undue reservation.

## Author contributions

EA, SH, FJ, MAK, MIm, FSa, AAH, MAA, and FSh designed the experiments. EA carried out the field trials and collected the data. SH, MIs, SK, HMA, WFAM, and FSh helped in data analysis and article preparation. All authors contributed to the article and approved the submitted version.
